# Quantitative Assessment of the Echogenicity of a Breast Tumor Predicts the Response to Neoadjuvant Chemotherapy

**DOI:** 10.3390/cancers13143546

**Published:** 2021-07-15

**Authors:** Katarzyna Sylwia Dobruch-Sobczak, Hanna Piotrzkowska-Wróblewska, Piotr Karwat, Ziemowit Klimonda, Ewa Markiewicz-Grodzicka, Jerzy Litniewski

**Affiliations:** 1 Ultrasound Department, Institute of Fundamental Technological Research, Polish Academy of Sciences, Pawińskiego 5B, 02-106 Warsaw, Poland; hpiotrzk@ippt.pan.pl (H.P.-W.); pkarwat@ippt.gov.pl (P.K.); zklim@ippt.pan.pl (Z.K.); jlitn@ippt.pan.pl (J.L.); 2 Radiology Department II, Maria Sklodowska-Curie National Research Institute of Oncology, 15 Wawelska St., 02-034 Warsaw, Poland; 3 Department of Oncology and Radiotherapy, Maria Sklodowska-Curie National Research Institute of Oncology, 15 Wawelska St., 02-034 Warsaw, Poland; ewa.mg@poczta.onet.pl

**Keywords:** quantitative ultrasound, B-mode ultrasound, echogenicity, breast cancer, neoadjuvant chemotherapy

## Abstract

**Simple Summary:**

B-mode US is a widely available, inexpensive, and non-invasive technique. This method is used in monitoring the neoadjuvant chemotherapy (NAC) in breast cancer (BC). In the presented study we combined the result from B-mode ultrasound examination with quantitative information about the characteristics and structure of the tissue in predicting the response to neoadjuvant chemotherapy in BC patients. We used echogenicity (ΔEcho) as B-mode features and the Kullback-Leibler divergence (ΔKLD) method as a quantitative parameter to provide information on changes in image echogenicity, to determine differences between the distributions of the ultrasound echo amplitude from tumor during NAC. The ΔKLD parameter alone is an accurate predictor of response to treatment after the second course of therapy (cut-off ≥70%, AUC = 0.85). Combining both parameters (ΔKLD and ΔEcho) led to an increase in sensitivity without significant deterioration of other statistical parameters and allowed to accurately predict non-responding tumors.

**Abstract:**

The aim of the study was to improve monitoring the treatment response in breast cancer patients undergoing neoadjuvant chemotherapy (NAC). The IRB approved this prospective study. Ultrasound examinations were performed prior to treatment and 7 days after four consecutive NAC cycles. Residual malignant cell (RMC) measurement at surgery was the standard of reference. Alteration in B-mode ultrasound (tumor echogenicity and volume) and the Kullback-Leibler divergence (kld), as a quantitative measure of amplitude difference, were used. Correlations of these parameters with RMC were assessed and Receiver Operating Characteristic curve (ROC) analysis was performed. Thirty-nine patients (mean age 57 y.) with 50 tumors were included. There was a significant correlation between RMC and changes in quantitative parameters (KLD) after the second, third and fourth course of NAC, and alteration in echogenicity after the third and fourth course. Multivariate analysis of the echogenicity and KLD after the third NAC course revealed a sensitivity of 91%, specificity of 92%, PPV = 77%, NPV = 97%, accuracy = 91%, and AUC of 0.92 for non-responding tumors (RMC ≥ 70%). In conclusion, monitoring the echogenicity and KLD parameters made it possible to accurately predict the treatment response from the second course of NAC.

## 1. Introduction

Breast cancer (BC) is a disease of significant social importance and is the most common malignant neoplasm in women in Poland and worldwide. The incidence of BC in women over the age of 30 is systematically increasing. In Poland in 2018, 18,869 BC cases were diagnosed in women and 154 in men [1].

The methods of treating BC, in particular systemic treatment, as well as the techniques used to monitor it, have changed significantly in recent years. Neoadjuvant chemotherapy (NAC), introduced in 1970, was initially used in locally advanced breast cancer (LABC) and in inflammatory BC to reduce the size of the tumor and improve the radicality of surgical treatment, including conserving breast conserving therapy (BCT). Currently, NAC is increasingly recommended in the early stages of BC, in the following subtypes: triple-negative cancer (TNBC) and with the presence of HER2 + receptors [2]. The goal of preoperative treatment is to achieve pathological complete response (pCR), which is a surrogate for overall survival (OS), event free survival (EFS), and long-term survival in TNBC and HER2 + subtypes [3].

The targeted HER2 + BC treatment regimen and new improved TNBC chemotherapy regimens significantly improved the pathology complete response (pCR) rate. However, the response to preoperative chemotherapy is variable, and complete tumor regression, confirmed by histopathology as pCR, occurs in an average of 19% of patients (range 0.3–50.3%, depending on the immunohistochemical subtype of BC) [4,5]. Approximately 20–30% of BCs remain insensitive to NAC, and chemotherapy delays the necessary surgical treatment, increases the risk of metastasis, and may contribute to side effects [3,4,5]. In addition, the largest group is that of partial responders, which is significantly heterogeneous, and as a result, the prediction of the precise rate of response is difficult. The most accurate method for monitoring treatment is a multi-parameter magnetic resonance imaging (MRI), especially in comparison to mammography (MMG) or ultrasonography (USG) [5]. In MRI, as in USG, the tumor size assessment according to RECIST 1.1 (response evaluation criteria in solid tumors) is not sufficiently sensitive in treatment monitoring due to the presence of necrotic lesions, which, although it responds well to treatment, does not change the size of the tumor [5]. Similarly, the assessment of the response to treatment based on the analysis of tumor vascularity in dynamic contrast-enhanced MRI (DCE-MRI) has several limitations that have been shown in a meta-analysis of 14 studies, with an average sensitivity and specificity of 84% and 83%, respectively [6]. However, in the meta-analysis assessing the diagnostic efficacy of CE-MMG (contrast-enhanced mammography) and CE-MRI, in the assessment of the response to NAC, CE-MMG was shown to be more sensitive and specificity was similar for both techniques [7].

For this reason, the introduction into common clinical practice of a non-invasive functional imaging system capable of monitoring the early tumor response to cancer therapy is a priority. Such an approach will enable the personalization of treatment of oncological patients and, consequently, will contribute to the optimization of the therapeutic outcome and relapse-free survival. It should be noted that the collection of data in the NAC monitoring studies is slow, laborious, and with varying incidence of certain types of cancer. This in turn limits the number and variety of tumors included in each study. In previous studies, the authors focused on a group of LABC patients. The current recommendations allow to qualify to NAC patients with early stage of BC with HER2 + and TNBC, which we included in our study [8,9].

Ultrasound imaging is a widely available, inexpensive, and non-invasive technique. The use of quantitative ultrasound (QUS) methods can supplement traditional imaging ultrasounds (considered to be a less objective method, as the test result largely depends on the quality of the equipment and the operator’s experience) with additional quantitative parameters [8,9]. These parameters are not based on a subjective assessment of changes in tissue echogenicity, but characterize its condition on the basis of information about its microstructure. Quantitative ultrasound is a tissue characterization technique that examines the “content” of ultrasound signals. According to the theory of ultrasound scattering, the backscattered signal returning from the tissue is influenced by parameters such as size, spatial distribution of the scatterers, and their properties.

Experiments using ultrasound data collected from cell pellets exposed to chemotherapeutics that induce apoptosis have shown a significant effect of apoptosis on ultrasound scattering and the echogenicity of ultrasound images [10,11]. These results encourage the use of Quantitative Ultrasound (QUS) in the context of monitoring the effects of chemotherapy.

The possibilities of using QUS methods in NAC monitoring have recently been widely explored. For example, Quiaoit et al. proposed a multi-parameter approach to the problem of detecting a positive response to therapy [12]. In this study, the authors generated parametric images using the spectral and backscattering parameters of the signals scattered within the tumor. Then they used the texture features of these images to develop multi-parametric models to predict pathological tumor response.

The best model was able to predict positive response to NAC with area under the ROC curve (AUC) value equal 0.87 after the first week of the therapy. Dasgupta et al. used textural features of QUS parametric images of spectral and scattering parameters of a tumor for predicting the response to NAC with AUC of 0.86 [13]. In other work, Tadayyon et al. showed the potential of combining QUS analysis with artificial neural networks as a source of diagnostic biomarkers [14]. The authors reported high performance of their model in predicting the pathological response (AUC = 0.96) and 5-year recurrence-free survival (RFS) of patients (AUC = 0.89) prior to the start of treatment. Despite high efficiency reported in those studies, there is still much room for improvement. Each new parameter that is reported to perform decent in predicting the response to NAC, is valuable, as it can potentially be included in a multi-parametric model, improving its performance.

In our study, we focused on the characterization of the tumor echogenicity, which is related to its structure and cellularity, as is the ADC in the MRI image [15,16]. In the classic B-mode examination, echogenicity is assessed in relation to adipose tissue, which makes the assessment more objective and independent of the ultrasonic scanner settings. The echogenicity of a given area of the examined tissue translates into the amplitude of the scattered signal and affects the average value of the amplitude distribution. Tissue signal amplitude distribution depends on tissue structure, and their mean values may differ depending on the tissue. Moreover, with a similar mean value, they can describe different tissue structures. Therefore, the differences between the distributions are more accurately determined using statistical measures of variation than by their mean values.

We used the Kullback-Leibler divergence (kld) method [17] to determine differences between the distributions of the ultrasound echo amplitude from tumor tissue undergoing successive cycles of NAC therapy, and, ultimately, to predict the effects of NAC. Amplitude distributions were determined from raw radio-frequency (RF) signals, which ensured independence from the processing methods used by scanners for image enhancement. At the same time, we investigated the possibility of predicting the effects of NAC using changes in B-mode image echogenicity and tumor size and the combination of both parameters. The results were assessed in relation to postoperative residual malignant cells (RMC) rate.

## 2. Materials and Methods

### 2.1. Patients

This prospective, single-center study was conducted in accordance with the Declaration of Helsinki, and the protocol was approved by the Ethics Committee of Maria Skłodowska–Curie National Institute of Oncology, Scientific Centre, Warsaw, Poland (Project identification code 49/2018). All participants provided informed consent for inclusion before participation in the study.

Breast ultrasound examinations were performed on 39 patients with a total of 50 BCs (seven women had bifocal lesions and two had trifocal lesions) from April 2016 to July 2020.

The inclusion criteria were as follows: inclusion in the NAC by an oncologist during multidisciplinary meeting (MDM); maximum tumor diameter <4 cm, minimum tumor diameter 5 mm; and multicenter ≤3 if in another quadrant and/or breast, regardless of the immunohistological subtype and lymph node status.

Ultrasound examinations were performed before NAC and 7 days after subsequent NAC courses (first to fourth). NAC was administered according to international guidelines, according to a previously detailed protocol [18]. AC (doxorubicin, cyclophosphamide) was used from the first to the fourth course, and then treatment was continued with taxol. Patients with HER2+ receptor-positive tumors were treated with trastuzumab and taxol. One patient with a history of contralateral BC 5 years prior to the study, was treated with AT (doxorubicin and docetaxel), 37 patients underwent mastectomy, 2 underwent BCT, 37 underwent lymphadenectomy, and 2 underwent sentinel lymph node dissection (SLND) at the end of chemotherapy. Four patients did not complete the study, which resulted in modification of the NAC data. All remaining patients underwent a full course of NAC therapy.

### 2.2. Histology

In all patients, core needle biopsies (CNB) were performed after administration of 2% lidocaine before qualification to NAC treatment (14GA diameter biopsy needle, three to five cores; Pro-Mag). An experienced pathologist with 25 years’ experience in breast oncological pathology assessed the cores from CNB and excisable tumors or residual intramammary target lesions with clip markers after NAC. Cancer subtypes (molecular subtypes and grade of malignancy) were obtained by pathological assessment after CNB. RMC rates in the residual malignant tumor were estimated by a pathologist based on postoperative histopathology and served as a reference of tumor response to NAC. RMC is one of the six features of the residual cancer burden (RCB) index that is a quantitative parameter evaluating the response to NAC that can be calculated using an on-line calculator. The percentage of RMC ranged from 0 to 100% and was determined after surgery [19]. In the statistical analysis, we evaluated the following cut-off values for RMC: ≤30% for responding tumors and ≥70% for non-responding tumors.

### 2.3. Ultrasonic Data Registration

The acquisition of RF ultrasound echoes from patients was performed using an Ultrasonix SonixTOUCH ultrasound scanner (Ultrasonix Medical Corporation, Richmond, BC, Canada). The breast lesions were assessed according to American College of Radiology (BI-RADS-lexicon) and the standards of Polish Ultrasound Society [20,21]. The scanner was equipped with an ultrasound research interface enabling the recording of raw post-beamformed RF data. The measurements were made using a linear probe (L14-5/38) at a frequency of 10 MHz. In accordance with the measurement protocol, the ultrasound RF data were recorded for each tumor in the following planes: radial, radial +45°, anti-radial, and anti-radial +45°. Classic amplitude images (B-mode) were generated from these data, with no additional post-processing applied. This is important because commercial scanners usually perform additional image processing to improve the images, for example, to increase contrast. Such operations influence the statistics of the image amplitudes to such a degree that they are difficult to assess. Therefore, in these studies, non-post-processed data were used.

Data registration was performed before the start of treatment and 7 days after each course of chemotherapy. In each B-mode image, a tumor was indicated by a radiologist with 20 years of experience in breast ultrasound. Data from four cross sections of the tumor were analyzed as a single set. This approach allowed for a more detailed analysis of changes in the tumor microstructure than was possible by examining only a single cross-section.

### 2.4. Quantitative Analysis of Ultrasound Data

**Figure 1:**
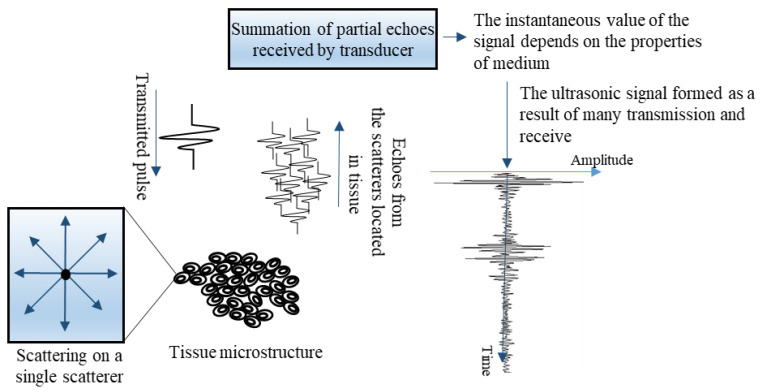
Diagram of ultrasonic radio frequency (RF) signal formation as a result of scattering on inhomogeneities present in the tissue.

The physical properties and spatial distribution of scatterers influence the statistical distribution of the amplitudes of the received echoes. A diagram of the ultrasonic signal generation is shown in Figure 1. Thus, the amplitude distribution of the ultrasonic signal received by the transducer contains information about the microstructure of the examined tissue. For example, if breast microcalcifications are present (a source of strong ultrasound scattering), higher amplitude values may be expected (Figure 1).

**Figure 2:**
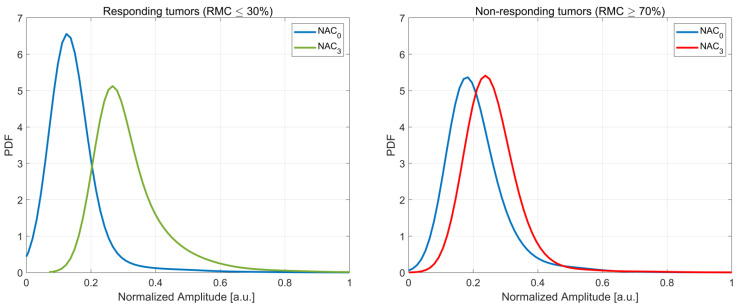
Average probability density (PDF) function for backscatter amplitude obtained for responders (RMC ≤ 30%) and non-responders (RMC ≥ 70%). Data were recorded before chemotherapy (NAC_0_) and after cycle 3 (NAC_3_).

Changes in the microstructure of the tumor occur as a result of the NAC effect. Remodeling is a multi-stage process, involving changes to cancer cells (cellular organization, cell death) and changes in the stroma. The degree of change in tumor depends on individual factors. However, large changes in the microstructure of responding tumors and small changes in tumors resistant to treatment with NAC can be expected. Therefore, it can be assumed that the degree of change in the amplitude distributions of the received signals is directly related to the degree of change at the microstructure level. This assumption forms the basis of the method presented in this study. The amplitude distributions were estimated using kernel density estimation (KDE) [22,23]. Amplitude distributions determined for responder (RMC ≤ 30%) and non-responder (RMC ≥ 70%) patients are shown in Figure 2, respectively.

The Kullback–Leibler divergence (kld) [13] was used to quantify the differences between two distributions of amplitudes (where *h_n_* and *h_m_* denote the amplitude distributions after the *n*-th and *m*-th treatment courses), which is given by the equation:

$$\text{kld}\left(h_{n},\;h_{m}\right)=\underset{i}{\sum }h_{n}\left(i\right)\log_{2}\frac{h_{n}\left(i\right)}{h_{m}\left(i\right)}$$

where *i* is a sample index in the amplitude distribution. The values of kld are always non-negative; a value of 0 indicates that the distributions *h_n_* and *h_m_* are identical. The kld was used to define three parameters:

$$KLD_{0}\left(n\right)=log_{10}\left[\text{kld}\left(h_{n},h_{0}\right)\right];\;\;n\in \left\{1,2,3,4\right\}$$

$$KLD_{1}\left(n\right)=log_{10}\left[\text{kld}\left(h_{n},h_{1}\right)\right];\;\;n\in \left\{2,3,4\right\}$$

$$\Delta KLD\left(n\right)=KLD_{0}\left(n\right)-KLD_{0}\left(1\right)=log_{10}\left[\frac{\text{kld}\left(h_{n},h_{0}\right)}{\text{kld}\left(h_{1},h_{0}\right)}\right];\;\;n\in \left\{2,3,4\right\}$$

where *h* is the estimated amplitude distribution, its subscript of 0 indicates pre-treatment data, and a positive subscript *n* indicates the NAC course at which the data were recorded. The use of logarithms in the above formulas results from highly asymmetric distributions of the KLD parameters if they are devoid of logarithm. After application of the logarithm, these distributions were close to normal.

To evaluate the effectiveness of NAC therapy, three quantitative parameters, all based on the KLD method, were used. The KLD_0_ parameter estimates the changes in the amplitude distributions in the tumor after particular NAC courses in relation to the distributions before the start of therapy. The KLD_1_ parameter describes the changes in amplitude distributions with respect to the amplitude distribution after the first NAC, and the ∆KLD parameter describes the differences between the distributions in relation to the difference between the distribution after the first NAC and the distribution before treatment initiation.

### 2.5. Tumor Echogenicity

Tumor echogenicity (Echo) was assessed by a physician based on gray-level standard B-mode images compared to fat tissue in the preglandular zones. The following echogenicity levels were assigned to the images of each tumor:

1. Hypoechoic
2. Hypo and isoechoic (mixed)
3. Isoechoic
4. Hyperechoic

The Echo change (ΔEcho) was determined in relation to pre-treatment value Echo(0) using the following equation:

ΔEcho(n)=Echo(n)−Echo(0);  n∈{1,2,3,4}

where *n* indicates the NAC course

### 2.6. Tumor Volume

The volume (V) of the tumor was calculated assuming an ellipsoidal shape using the following equation:

$$V=\frac{4\pi }{3}\times \frac{w}{2}\times \frac{d}{2}\times \frac{h}{2}$$

where *w*, *d*, and *h* represent the width, depth, and height of the tumor, respectively, assessed based on the B-mode images. The volume change (ΔV) was determined in relation to pre-treatment value V(0) using the following formula:

$$\Delta V\left(n\right)=log_{10}\left[\frac{V\left(n\right)}{V\left(0\right)}\right];\;\;n\in \left\{1,2,3,4\right\}$$

where *n* indicates the NAC course. For the same reason as KLD, ΔV is subjected to logarithm. In order to enable an intuitive evaluation of volume changes, a percentage change in volume ΔV_%_ was defined as:

$$\Delta V_{\%}\left(n\right)=\left[\frac{V\left(n\right)}{V\left(0\right)}-1\right]\times 100\%;\;\;n\in \left\{1,2,3,4\right\}$$

### 2.7. Statistical Analysis

All of the considered parameters were examined in terms of their correlation with the RMC at particular stages of therapy. As none of the abovementioned parameters met the normality criterion at all stages of the therapy (Shapiro–Wilk test, significance level 0.05), the Spearman’s rank correlation coefficient r_S_ was used to assess the correlation. Subsequently, individual parameters were analyzed in the context of tumor classification as “responding” and “non-responding.” Tumors with RMC ≤ 30% were considered responders, and those with RMC ≥ 70% were considered non-responders. Each of the above parameters was analyzed as a single-parameter classifier. In addition, the ΔEcho and ΔV parameters were combined in a two-parameter classifier using linear discriminant analysis (LDA), which is a generalization of Fisher’s linear discriminant [24]. The tested classifiers were cross-validated using the leave-one-out (LOO) method [25]. The only exception was the classifier based solely on ΔEcho, where, because of the coarse discretization of the parameter, the cross-validation algorithm led to a distortion of the analysis results and was therefore omitted.

The effectiveness of the classification was assessed based on the receiver operating characteristics (ROC) curve, which demonstrated a trade-off between the true positive rate (TPR) and the false positive rate (FPR) for a classifier. The area under the ROC curve (AUC) was used for an overall assessment of the entire ROC curve [26]. For a random classifier, the AUC is close to 0.5, whereas for the ideal classifier, the AUC is equal to 1. The confidence intervals for the AUC were determined using the bootstrap method. The number of bootstrap samples was 10^4^, and the confidence level was 0.95. Each classifier was assessed in terms of its sensitivity, specificity, accuracy, positive predictive value (PPV), and negative predictive value (NPV). These features were determined for the optimal operating point, which was chosen as the point on the classifier’s ROC curve closest to the point (FPR = 0, TPR = 1) in the Euclidean sense [27].

## 3. Results

The mean age of the patients was 57 years (range, 32–83 years; median, 56 years; SD, 15). Histopathological verification before surgery revealed that the tumors comprised invasive carcinoma NST G2 (22 tumors), G3 (9 tumors), and G1 (11 tumors). Moreover, there were 9 luminal A cancers, 24 luminal B, 9 TNBC, and 8 HER2^+^ tumors. In our group of patients, the structure of the breasts was as follows: glandular (*n* = 9), fatty (*n* = 11) or mixed (*n* = 19).

The clinical details of the patients are shown in Table 1. Histopathological examination after final NAC and surgery revealed 28 tumors with 0–30% RMC, including 14 tumors with RMC = 0 (pathological complete response pCR), 9 tumors with RMC of 31%–69%, and 13 with RMC ≥ 70% (Table 1).

### 3.1. Tumor Echogenicity

**Figure 3:**
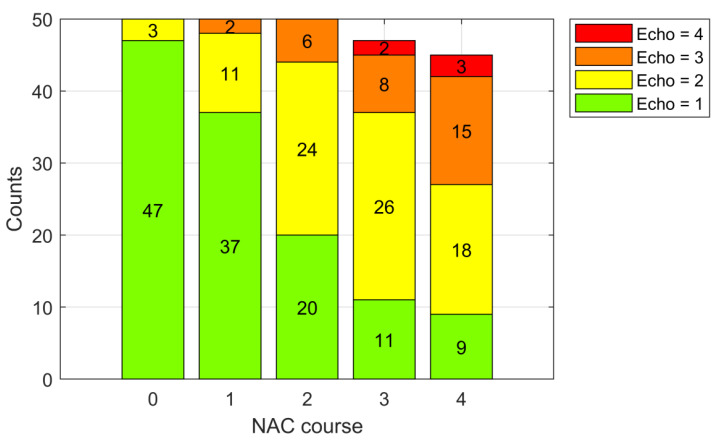
Echogenicity distribution of tumors before treatment (0) and after subsequent chemotherapy courses.

Before treatment, 47 tumors were hypoechoic, and three presented mixed echogenicity. After three courses of NAC, only 11 of the remaining 47 tumors were hypoechogenic, 26 presented mixed echogenicity, 8 were isoechogenic, and 2 were hyperechogenic (Figure 3).

**Figure 4:**
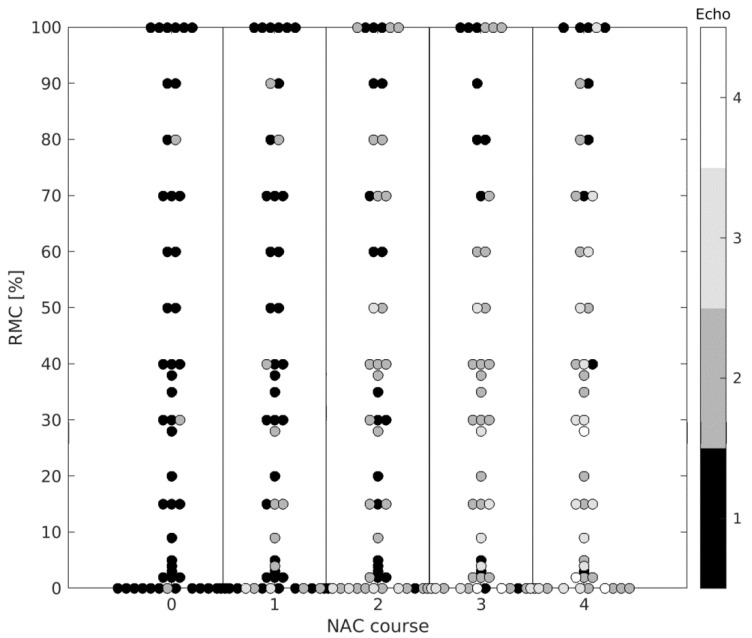
Alteration of echogenicity (Echo) after subsequent courses of NAC in reference to RMC assessed after treatment.

Before treatment, the average RMC calculated for 47 hypoechogenic tumors (Echo = 1) was 35%, compared with 57% after three courses of NAC (*n* = 11) and 76% after four courses of NAC (*n* = 9). In tumors that become isoechogenic (Echo = 3), the average RMC value was 13% after three courses of NAC (*n* = 8) and 28% after four courses of NAC (*n* = 15) (Figure 3 and Figure 4, Table 2).

### 3.2. Tumor Size

**Figure 5:**
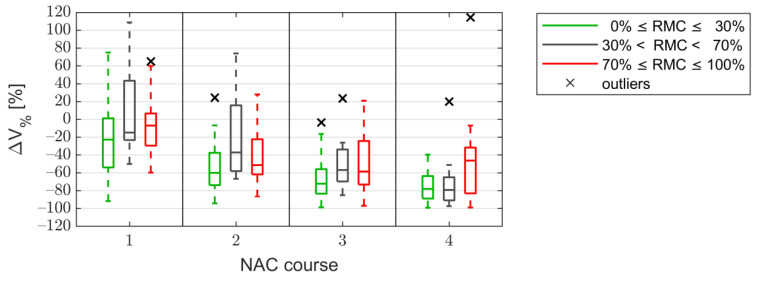
Statistics of the volume change ΔV_%_ after subsequent courses of neoadjuvant chemotherapy (NAC). The tumors are divided into responding (RMC ≤ 30%), partially-responding (30% < RMC < 70%), and non-responding (RMC ≥ 70%). (RMC—Residual Malignant Cell).

The mean size of the lesion before the treatment (*n* = 50) was 5.0 cm^3^ (median, 2.8 cm^3^; range, 0.04–27 cm^3^; SD, 5.7 cm^3^), and after three courses of NAC (*n* = 47) it was 2.0 cm^3^ (median, 0.67 cm^3^; range, 0.02–14 cm^3^; SD, 2.8 cm^3^). When analyzing changes in the volume of neoplastic tumors, decreases were observed in the entire group of tumors after the first and second courses of NAC, while a reversal of this trend was observed after the third course of NAC. For tumors that responded poorly to treatment (RMC ≥ 70%), an increase in tumor volume was observed in relation to the previous courses, although they were still smaller than the pre-treatment measurement (Figure 5).

### 3.3. The Kullback–Leibler Divergence Based Parameters

**Figure 6:**
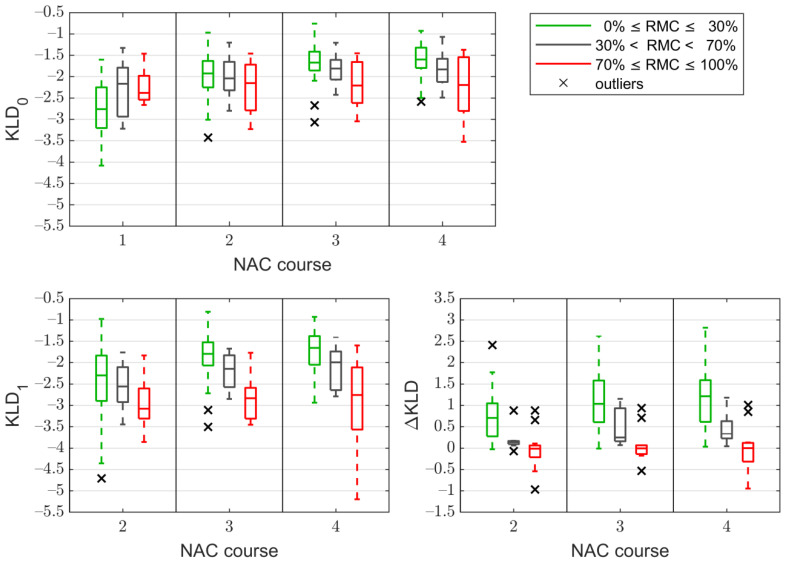
Statistics of the kld-based parameters: KLD_0_ (top), KLD_1_ (bottom left), and ΔKLD (bottom right), after subsequent courses of neoadjuvant chemotherapy (NAC). The tumors are divided into responding (RMC ≤ 30%), partially-responding (30% < RMC < 70%), and non-responding (RMC ≥ 70%) (RMC—Residual Malignant Cell).

The statistics of the Kullback–Leibler divergence (kld)—related parameters are shown in Figure 6. Distributions of each parameter show improvement in the separation of responders and non-responders with subsequent courses of NAC. The best separation is observed for the ΔKLD parameter.

### 3.4. Correlation

**Figure 7:**
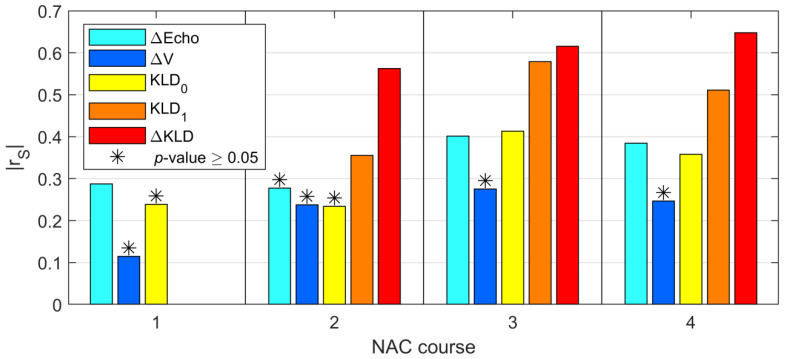
Correlation values for the five analyzed parameters (ΔEcho, ΔV, KLD_0_, KLD_1_, and ΔKLD) as a function of chemotherapy courses. Statistical insignificance (*p*-value ≥ 0.05) marked with asterisks.

Prior to analyzing the usefulness of the parameters as predictors of the efficacy of NAC, their correlations with the RMC results were assessed. After the first course of NAC, the ΔEcho, ΔV, and KLD_0_ parameters showed weak and mostly statistically insignificant correlations, which only slightly improved with subsequent NAC courses. The KLD_1_ and ΔKLD parameters, which were available starting from the second course of NAC, demonstrated statistically significant, mostly moderate or strong correlations (Figure 7).

A table demonstrating the resulting Spearman correlations and *p*-values is available in Appendix A (Table A1).

### 3.5. Classification

**Figure 8:**
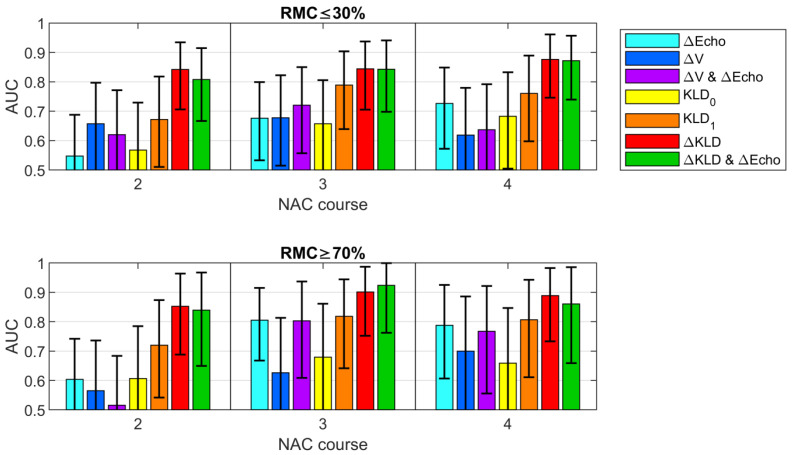
AUC values determined for the examined classifiers as a function of chemotherapy courses for two thresholds: RMC ≤ 30% and RMC ≥ 70%.

**Figure A1:**
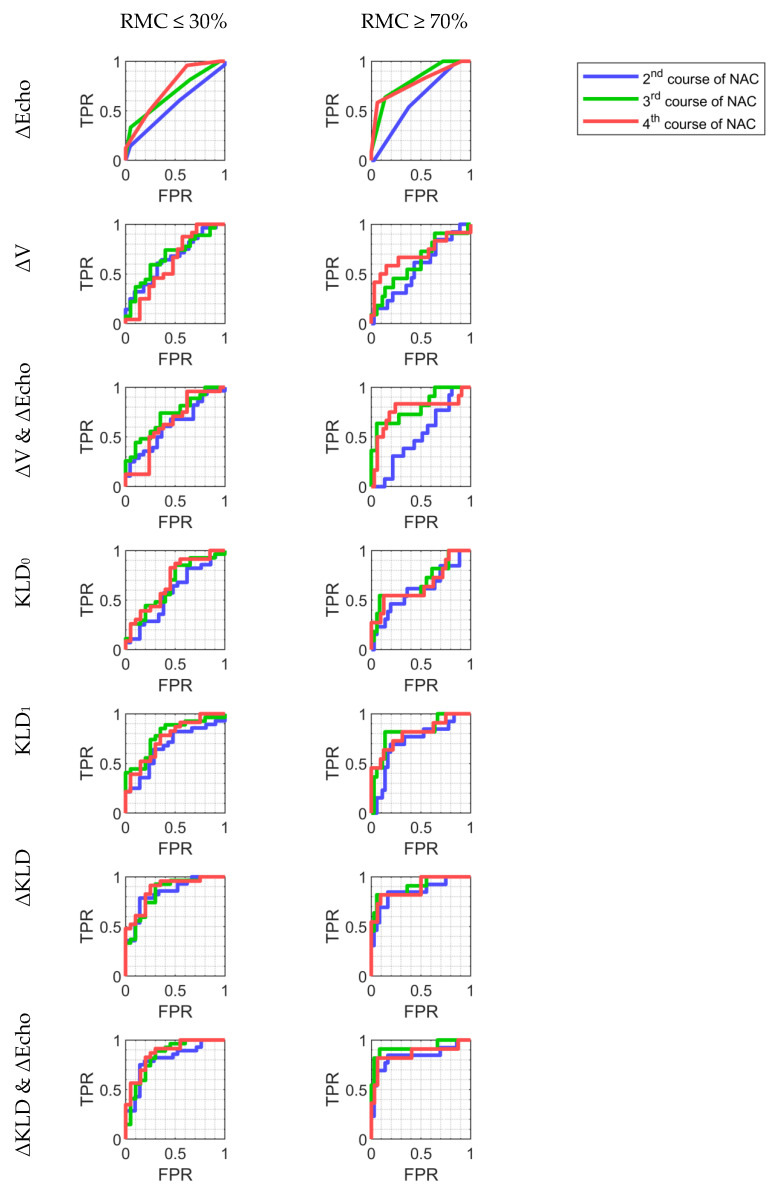
ROC curves for the examined classifiers and four RMC thresholds.

Two RMC thresholds (RMC ≤ 30% and RMC ≥ 70%) were analyzed in order to divide the tumors into “responder” and “non-responder” groups. Figure 8 shows the AUC values together with confidence intervals for the analyzed parameters, as a function of chemotherapy courses. The corresponding ROC curves are included in the Appendix A (Figure A1). The Appendix A also includes complete tables of classification performance of responders and non-responders (Table A2 and Table A3, respectively). Their essential parts are presented in the main body of the article as Table 3, Table 4 and Table 5, for later discussion.

Of the B-mode parameters, assessed separately and in combination, the assessment of changes in echogenicity (ΔEcho) most accurately predicted the group of non-responding tumors with RMC ≥ 70% (Table 3) after the third NAC course (AUC = 0.81), with a sensitivity of 64% and specificity of 86%. To evaluate the volume change (ΔV), lower AUC values were obtained after both the third (AUC = 0.63) and fourth (AUC = 0.79) courses for RMC ≥ 70%.

We found that there was a slight improvement in accuracy, specificity, and PPV with the combined model of both parameters (change in echogenicity and volume) (accuracy 87% vs. 80%, specificity 94% vs. 86%, 58% vs. 78%), with no improvement in sensitivity (64%) (Table 5). The results for all of the RMC cutoff values are shown in Appendix A (Table A2 and Table A3).

**Figure 9:**
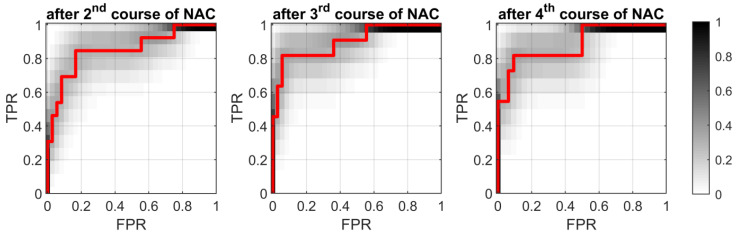
ROC curves (with marked grayscale probability density values) determined for the ΔKLD parameter after the second, third, and fourth courses of chemotherapy for RMC exceeding 70%.

Starting from the second course of chemotherapy, the quantitative parameter ΔKLD made it possible to predict the response to treatment with AUC ≥ 0.85. The ROC curves obtained after the second, third, and fourth courses of chemotherapy are presented in Figure 9. As in the case of echogenicity, the ΔKLD brought the best results after the third treatment course (AUC = 0.90; sensitivity of 82%; specificity of 94%). All of the classifiers characteristics are listed in Table 3.

**Figure 10:**
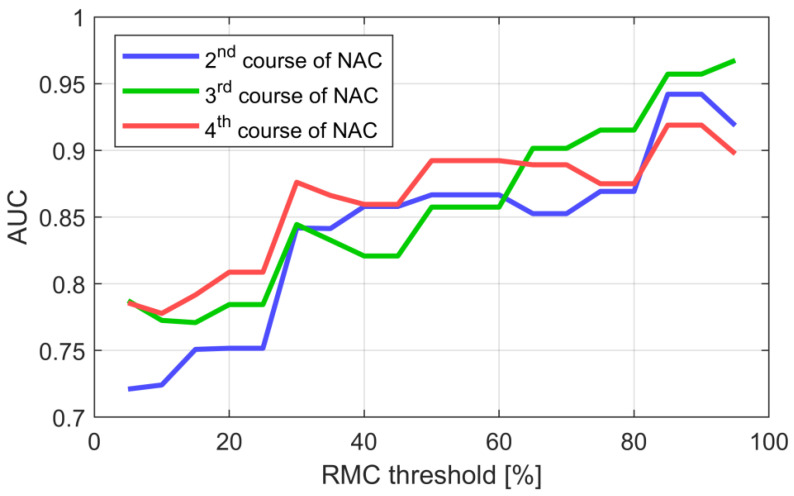
AUC of the ΔKLD-based classifier as a function of the adopted RMC threshold level.

The influence of the selection of the RMC threshold on the efficiency of the ΔKLD-based classifier is shown in Figure 10. The AUC increases with the adopted RMC threshold, which means that non-responding tumors can be indicated more efficiently. Detection of the tumors with the worst response to NAC (RMC ≥ 90%) is characterized with AUC significantly exceeding 0.90. While the classification of responders with use of the ΔKLD parameter appears more difficult, it still allows to achieve AUC = 0.84 for RMC ≤ 30% (Table 4).

## 4. Discussion

In the current study, ultrasound images were analyzed to monitor the response to NAC after the first four courses. Two types of images were included: traditional B-mode images and amplitude images generated from reconstructed (post-beamformed) RF data. The purpose of using the RF data was to avoid the unknown impact of image post-processing algorithms implemented in commercial scanners. 

In traditional B-mode images, changes in volume and echogenicity were analyzed relative to the baseline study before treatment. Quantitative ultrasonic examination was based on the KLD analysis. Three parameters, KLD_0_, KLD_1_, and ΔKLD, were used to assess the discrepancy between the distributions of echogenicity of the images after particular NAC courses compared to the distributions for amplitude images determined: (1) before the start of treatment and (2) after the first treatment course.

In the B-mode assessment, we showed that the change in tumor volume following subsequent courses of NAC is not an accurate parameter when analyzed independently; however, in combination with the change in echogenicity, it improved the AUC (0.8 vs. 0.63) and specificity (up to 94%), although with no improvement in sensitivity (64%) after the third NAC course for the non-responding tumors (for RMC ≥ 70%). However, the necrotic areas within the tumor are likely to represent a limitation of this parameter. These areas appear as hypoechoic and they do not affect the change in tumor volume assessed in the B-mode study, which may falsely indicate a lack of response to treatment. However, in studies using MRI in monitoring the response to NAC in BC, Minarikowa et al. showed that, as in the ultrasound evaluation, the appearance of necrosis led to false results [28]. A study showed that higher ADC values before treatment are associated with the presence of necrosis and limited perfusion, and a limited response to treatment should be expected in these tumors as a result. Chu et al. showed that the increase in the ADC parameter value predicts the pCR with a sensitivity of 88% and a specificity of 79% [29]. ADC is a quantitative parameter that measures diffusivity derived from diffusion-weighted imaging (DWI), which is a non-contrast method that evaluates water mobility and tissue cellularity. Increasing ADC values during NAC therapy reflect increased cell lysis and necrosis [30].

Marinovich et al. showed in a meta-analysis that the assessment of the changes in tumors size after NAC assessed by ultrasound and MRI indicates an underestimation of the size of tumors in the ultrasound examination; however, the MRI examination has a tendency to overestimate the size of the residual tumor in relation to histopathological verification [30]. The RECIST criteria used in MRI are not recommended for the assessment of changes in tumor size in ultrasound examination [31,32]. In this study, authors showed that US examination was operator-dependent and characterized by low repeatability. On the other hand, for the next parameter in the B-mode assessment, echogenicity, we showed statistically significant correlation with the RMC value after the third and fourth NAC courses. For the classification of non-responders (RMC ≥ 70%), after the third NAC course, AUC, specificity, and sensitivity were 0.81, 86%, and 64% respectively.

For comparison, in a study on 42 focal lesions, Dobruch-Sobczak et al. analyzed changes in echogenicity after the third NAC course, assuming that tumors did not respond if RMC values were ≥70% and showed sensitivity of 73%; specificity of 87%; PPV of 67%; NPV of 90%; accuracy of 83%; and AUC = 0.69. In the same group, in two parameters analysis using echogenicity and stiffness, an improvement in the statistical parameters was obtained: sensitivity of 82%, specificity of 90%, PPV of 75%, NPV of 93%, accuracy of 88%, and AUC of 0.88. It was noticed that both the stiffness and the hypoechoic nature of the lesions remained unchanged [15].

Therefore, based on the results of statistical analysis, we can assume that changes only in tumor echogenicity, for the group of tumors with RMC ≤ 30%, do not allow the correct assessment of the effects of NAC. In this group, the distinction between residual tumor and fibrosis in the tumor stroma is not possible by analyzing the variability of echogenicity assessed in relation to adipose tissue in the classic B-mode examination. Changes in the residual tumor cells, if present, are variable in pathology examination. More commonly, in microscopic analysis, carcinomas become less cellular and often present as small scattered nests across the tumor bed. After a complete response, only the oedematous vascularized fibroelastotic area of connective tissue with chronic inflammatory cells and macrophages mark the tumor bed [33]. Before treatment, the tumors are rich in malignant cellularity and transmit RF sound better than surrounding breast tissue, and are predominantly hypoechogenic. In contrast, in tumors resistant to NAC, no morphological alteration could be observed, and therefore, the echogenicity was unchanged.

In ultrasound diagnostics, the echogenicity of the B-mode image is a qualitative parameter, even if it is assessed in comparison to the adipose tissue. Although this approach significantly reduces the influence of the ultrasonic scanner settings on the assessment of echogenicity, it remains a subjective assessment by the physician. Image echogenicity is strictly related to the amplitude of the ultrasonic signal scattered in the tissue and the distribution of this amplitude. In the selected area, the assessment of echogenicity is largely dependent on the mean amplitude of the signal. Therefore, the same echogenicity values can be assigned to different amplitude distributions. In our research, we used the KDE (Kernel Density Estimation) method to describe the amplitude distribution of images determined from RF signals, and the KLD (Kullback–Leibler Divergence) method to detect differences between the amplitude distributions of tumor images in subsequent cycles of NAC. The method proposed in this study makes it possible to differentiate the amplitude distributions even when they have the same mean value, which, in relation to the average echogenicity, also enables better tracking of changes taking place in the tissue. Additionally, KLD assessment overcomes the limitations of the subjective assessment of echogenicity based on B-mode images.

Changes in the amplitude distributions in the tumor after particular courses of treatment and the distribution before treatment (KLD_0_) did not significantly correlate with the RMC for the first and second cycles of NAC, and after the subsequent cycles, the correlation was low (0.3–0.4), but statistically significant. This was reflected during the classification of tumors, which translated into low values of the classification parameters (see Table A3 in Appendix A). Compared to KLD_0_, the KLD_1_ parameter correlated better with the RMC.

Applying KLD_1_ to predict a poor tumor response (RMC ≥ 70%), the AUC values after the second, third, and fourth NAC cycles were 0.72, 0.82, and 0.81, respectively. The best results were achieved using ΔKLD. After the second course, NAC was able to indicate the non-responding tumors with sensitivity of 84%, specificity of 83%, accuracy of 84%, NPV of 94%, and AUC = 0.85. These values were even higher during the next stages of treatment. The best values after the third treatment course were 82%, 94%, 91%, 94%, and 0.90, respectively. For comparison, the assessment of the effectiveness of treatment based on changes in echogenicity in the B-mode examination allowed us to obtain accuracy, specificity, and NPV exceeding 80% after the third NAC course; however, these values were lower than those obtained for the ΔKLD parameter. This trend was unchanged after the fourth NAC treatment.

In the statistical analysis combining both parameters (ΔKLD and ΔEcho) after the second NAC course, all parameters describing the ability to classify remained at the same level as those for classification, which was based only on the ΔKLD parameter. After the third course of NAC, an increase in sensitivity was obtained from 82% to 91%, with a slight decrease in specificity from 94% to 92%, and the values of accuracy and AUC remained at the same high level of 0.91. It is worth noting that this parameter was also a predictor with high potential for the RMC cut-off value of ≤30%. After the second and third courses of NAC, the AUC values obtained were 0.83 and 0.84, respectively. These results suggest that the use of ΔKLD has great potential as a tool for predicting treatment response to NAC.

Note that ΔKLD actually uses two parameters, KLD_0_(n) and KLD_0_(1). Such effective operation of the classification with the use of ΔKLD results from the value of the KLD_0_(1) parameter. The values of this parameter, as shown in the Figure 5, after the first NAC cycle, are higher for tumors with RMC ≥ 70% than for tumors with RMC ≤ 30%. The KLD_0_(1) parameter is in the denominator of the formula describing the ΔKLD parameter. As a result, dividing by KLD_0_(1) results in an additional elevation of the ΔKLD value for non-responders compared to responders, which improves the classification.

We acknowledge that our study may have some weakness. In this research, post-beamformed RF data were used to avoid the unknown impact of image post-processing algorithms implemented in commercial scanners. RF data are not directly available in common scanners. However, they are always present in the data processing pipeline, and thus QUS methods can be potentially easily implemented on new US platforms.

We only used RMC as a reference standard, which is one of the six residual cancer burdens (RCB) assessed by the pathologist, but in the publication the most common trait referenced by many authors is tumor cellularity.

Another limitation of the study is that only one medical doctor assessed all ultrasound images, however, she has worked for 21 years at the Institute of Oncology and has extensive experience in breast imaging. In B-mode examination, we followed ACR BIRADS-lexicon.

## 5. Conclusions

In this study, evaluating the response of BC to NAC after the first four treatment courses, the ΔKLD parameter, which provides quantitative information on changes in image echogenicity, is an accurate predictor of poor response to treatment (RMC ≥ 70%) after the second course of therapy. In the statistical analysis combining ΔKLD and ΔEcho, an increase in sensitivity was obtained without significant deterioration of other statistical parameters.

Our research demonstrates that alterations in tumor echogenicity during NAC treatment are important features in assessing the therapy outcome. The evaluation of changes in echogenicity on the basis of statistical measures applied to the amplitude distributions of the ultrasound image is a particularly promising method. This approach makes it possible to predict the pathological response after NAC. The number of cases used in our research is a limitation in making more general conclusions. Nevertheless, we believe that our results show an important role of quantitative ultrasound in predicting the effects of chemotherapy in breast cancer patients.

## Figures and Tables

**Table 1:** Characteristic of the patients group, including histological findings.

Category	Characteristic	Count/Value
Patients	Number of patients	39
	Mean age (years)	57
	Age range (years)	32–83
Tumor histology	Invasive ductal carcinoma (IDC)	50
	IDC with ductal carcinoma in situ	20
Receptor status	Luminal A	9
	Luminal B	24
	TNBC	9
	HER 2+	8
Pathological response (RMC%)	0	14
	≤30	28
	31–69	9
	≥70	13
Surgical treatment	Mastectomy	37
Surgical treatment	BCT	2

BCT—breast conserving therapy, RMC—Residual Malignant Cell, TNBC—triple-negative breast cancer, HER2+—human epidermal growth factor receptor.

**Table 2:** Average (μRMC) and standard deviation (σRMC) of RMC in relation to echogenicity after successive courses of NAC.

Echogenicity	Pre-Treatment			1st NAC Course			2nd NAC Course			3rd NAC Course			4th NAC Course		
	*n*	μ_RMC_	σ_RMC_	*n*	μ_RMC_	σ_RMC_	*n*	μ_RMC_	σ_RMC_	*n*	μ_RMC_	σ_RMC_	*n*	μ_RMC_	σ_RMC_
Echo = 1	47	35	37	37	40	37	20	41	39	11	57	45	9	76	34
Echo = 2	3	37	40	11	26	32	24	38	36	26	34	32	18	28	31
Echo = 3	0	-	-	2	0	0	6	8	20	8	13	18	15	28	30
Echo = 4	0	-	-	0	-	-	0	-	-	2	0	0	3	10	16
All	50	35	36	50	35	36	50	35	36	47	34	36	45	37	36

**Table 3:** Results of statistical analysis of changes in echogenicity, volume, and ΔKLD parameter after two, three, and four courses of NAC for non-responding tumors (RMC ≥ 70%).

Measure of Performance	2nd NAC Course						3rd NAC Course						4th NAC Course					
	ΔEcho		ΔV		ΔKLD		ΔEcho		ΔV		ΔKLD		ΔEcho		ΔV		ΔKLD	
AUC	0.60	0.75	0.57	0.74	0.85	0.96	0.81	0.91	0.63	0.80	0.90	0.99	0.79	0.92	0.70	0.88	0.89	0.99
		0.46		0.37		0.69		0.66		0.42		0.75		0.60		0.48		0.73
Sensitivity	0.54		0.62		0.85		0.64		0.73		0.82		0.58		0.67		0.82	
Specificity	0.62		0.57		0.83		0.86		0.50		0.94		0.94		0.73		0.91	
Accuracy	0.60		0.58		0.84		0.81		0.55		0.91		0.84		0.71		0.88	
PPV	0.33		0.33		0.65		0.58		0.31		0.82		0.78		0.47		0.75	
NPV	0.79		0.81		0.94		0.89		0.86		0.94		0.86		0.86		0.94	

**Table 4:** Results of statistical analysis for changes in echogenicity, volume, and ΔKLD parameter after two, three and four courses of NAC for responders (RMC ≤ 30%).

Measure of Performance	2nd NAC Course						3rd NAC Course						4th NAC Course					
	ΔEcho		ΔV		ΔKLD		ΔEcho		ΔV		ΔKLD		ΔEcho		ΔV		ΔKLD	
AUC	0.55	0.69	0.66	0.8	0.84	0.93	0.68	0.8	0.68	0.82	0.84	0.94	0.73	0.85	0.62	0.78	0.88	0.96
		0.4		0.5		0.71		0.53		0.51		0.71		0.57		0.44		0.75
Sensitivity	0.61		0.61		0.79		0.33		0.74		0.93		0.5		0.75		0.91	
Specificity	0.45		0.68		0.86		0.95		0.6		0.7		0.76		0.48		0.75	
Accuracy	0.54		0.64		0.82		0.6		0.68		0.83		0.62		0.62		0.84	
PPV	0.59		0.71		0.88		0.9		0.71		0.81		0.71		0.62		0.81	
NPV	0.48		0.58		0.75		0.51		0.63		0.88		0.57		0.63		0.88	

**Table 5:** Combined model for changes in echogenicity and volume after individual NAC courses for RMC ≥ 70%.

RMC ≥ 70%	1st NAC Course	2nd NAC Course	3rd NAC Course	4th NAC Course
AUC	0.62	0.52	0.8	0.77
Sensitivity	0.69	0.62	0.64	0.83
Specificity	0.68	0.43	0.94	0.76
Accuracy	0.68	0.48	0.87	0.78
PPV	0.43	0.28	0.78	0.56
NPV	0.86	0.76	0.89	0.93

## Data Availability

Data supporting reported results can be found in Ultrasound Department, Polish Academy of Science.

## Appendix A

**Table A1:** Spearman correlations and *p*-values for assessed parameters after first: second, third and fourth courses of NAC.

Parameter	1st NAC Course	2nd NAC Course	3rd NAC Course	4th NAC Course
ΔEcho	−0.29 (0.043)	−0.28 (0.051)	−0.40 (0.005)	−0.38 (0.009)
ΔV	+0.11 (0.427)	+0.24 (0.096)	+0.28 (0.061)	+0.25 (0.102)
KLD_0_	+0.24 (0.095)	−0.23 (0.106)	−0.41 (0.004)	−0.36 (0.018)
KLD_1_		−0.36 (0.012)	−0.58 (2 × 10^−5^)	−0.51 (5 × 10^−4^)
ΔKLD		−0.56 (3 × 10^−5^)	−0.62 (4 × 10^−6^)	−0.65 (3 × 10^−6^)

**Table A2:** Statistical analysis for all measured US parameters (single ΔEcho, ΔV, KLD_0_, and KLD_1_, and their combinations ΔEcho with ΔV and ΔEcho with ΔKLD and in combination) assessed after first, second, third, fourth courses od NAC for RMC ≤ 30%.

Stage of Treatment	Parameter	AUC		Sensitivity	Specificity	Accuracy	PPV	NPV
1st NAC course	ΔEcho	0.62	0.73 0.5	0.36	0.91	0.6	0.83	0.53
	ΔV	0.65	0.79 0.49	0.64	0.73	0.68	0.75	0.62
	ΔV & ΔEcho	0.68	0.82 0.52	0.71	0.68	0.7	0.74	0.65
	KLD_0_	0.69	0.82 0.53	0.54	0.86	0.68	0.83	0.59
	KLD_1_	-	- -	-	-	-	-	-
	ΔKLD	-	- -	-	-	-	-	-
	ΔKLD & ΔEcho	-	- -	-	-	-	-	-
2nd NAC course	ΔEcho	0.55	0.69 0.4	0.61	0.45	0.54	0.59	0.48
	ΔV	0.66	0.8 0.5	0.61	0.68	0.64	0.71	0.58
	ΔV & ΔEcho	0.62	0.76 0.45	0.61	0.64	0.62	0.68	0.56
	KLD_0_	0.57	0.73 0.4	0.57	0.62	0.59	0.67	0.52
	KLD_1_	0.67	0.82 0.51	0.64	0.71	0.67	0.75	0.6
	ΔKLD	0.84	0.94 0.71	0.79	0.86	0.82	0.88	0.75
	ΔKLD & ΔEcho	0.81	0.92 0.66	0.79	0.81	0.8	0.85	0.74
3rd NAC course	ΔEcho	0.68	0.8 0.53	0.33	0.95	0.6	0.9	0.51
	ΔV	0.68	0.82 0.51	0.74	0.6	0.68	0.71	0.63
	ΔV & ΔEcho	0.72	0.85 0.55	0.74	0.65	0.7	0.74	0.65
	KLD_0_	0.66	0.81 0.49	0.85	0.5	0.7	0.7	0.71
	KLD_1_	0.79	0.9 0.63	0.74	0.75	0.74	0.8	0.68
	ΔKLD	0.84	0.94 0.7	0.93	0.7	0.83	0.81	0.88
	ΔKLD & ΔEcho	0.84	0.94 0.7	0.81	0.75	0.79	0.81	0.75
4th NAC course	ΔEcho	0.73	0.85 0.58	0.5	0.76	0.62	0.71	0.57
	ΔV	0.62	0.78 0.44	0.75	0.48	0.62	0.62	0.63
	ΔV & ΔEcho	0.64	0.79 0.46	0.58	0.67	0.62	0.67	0.58
	KLD_0_	0.68	0.83 0.5	0.83	0.55	0.7	0.68	0.73
	KLD_1_	0.76	0.88 0.6	0.78	0.65	0.72	0.72	0.72
	ΔKLD	0.88	0.96 0.75	0.91	0.75	0.84	0.81	0.88
	ΔKLD & ΔEcho	0.87	0.95 0.74	0.83	0.8	0.81	0.83	0.8

**Table A3:** Statistical analysis for all measured US parameters (single ΔEcho, ΔV, KLD_0_, and KLD_1_, and their combinations ΔEcho with ΔV and ΔEcho with ΔKLD and in combination) assessed after first, second, third, fourth courses od NAC for RMC ≥ 70%.

Stage of treatment	Parameter	AUC		Sensitivity	Specificity	Accuracy	PPV	NPV
1st NAC course	ΔEcho	0.6	0.7 0.48	0.92	0.3	0.46	0.32	0.92
	ΔV	0.6	0.77 0.43	0.69	0.62	0.64	0.39	0.85
	ΔV & ΔEcho	0.62	0.78 0.45	0.69	0.68	0.68	0.43	0.86
	KLD_0_	0.67	0.8 0.5	0.92	0.51	0.62	0.4	0.95
	KLD_1_	-	- -	-	-	-	-	-
	ΔKLD	-	- -	-	-	-	-	-
	ΔKLD & ΔEcho	-	- -	-	-	-	-	-
2nd NAC course	ΔEcho	0.6	0.74 0.45	0.54	0.62	0.6	0.33	0.79
	ΔV	0.57	0.73 0.38	0.62	0.57	0.58	0.33	0.81
	ΔV & ΔEcho	0.52	0.69 0.35	0.62	0.43	0.48	0.28	0.76
	KLD_0_	0.61	0.79 0.41	0.62	0.64	0.63	0.38	0.82
	KLD_1_	0.72	0.87 0.54	0.69	0.81	0.78	0.56	0.88
	ΔKLD	0.85	0.96 0.68	0.85	0.83	0.84	0.65	0.94
	ΔKLD & ΔEcho	0.84	0.97 0.65	0.85	0.83	0.84	0.65	0.94
3rd NAC course	ΔEcho	0.81	0.92 0.67	0.64	0.86	0.81	0.58	0.89
	ΔV	0.63	0.8 0.42	0.73	0.5	0.55	0.31	0.86
	ΔV & ΔEcho	0.8	0.94 0.62	0.64	0.94	0.87	0.78	0.89
	KLD_0_	0.68	0.86 0.47	0.55	0.92	0.83	0.67	0.87
	KLD_1_	0.82	0.95 0.64	0.82	0.86	0.85	0.64	0.94
	ΔKLD	0.9	0.99 0.75	0.82	0.94	0.91	0.82	0.94
	ΔKLD & ΔEcho	0.92	1 0.76	0.91	0.92	0.91	0.77	0.97
4th NAC course	ΔEcho	0.79	0.92 0.61	0.58	0.94	0.84	0.78	0.86
	ΔV	0.7	0.88 0.48	0.67	0.73	0.71	0.47	0.86
	ΔV & ΔEcho	0.77	0.92 0.56	0.83	0.76	0.78	0.56	0.93
	KLD_0_	0.66	0.84 0.43	0.55	0.88	0.79	0.6	0.85
	KLD_1_	0.81	0.95 0.62	0.73	0.78	0.77	0.53	0.89
	ΔKLD	0.89	0.98 0.73	0.82	0.91	0.88	0.75	0.94
	ΔKLD & ΔEcho	0.86	0.98 0.66	0.82	0.94	0.91	0.82	0.94
